# Targeting Metabolic Dysfunction-Associated Steatotic Liver Disease (MASLD): Available and Future Pharmaceutical Options

**DOI:** 10.7759/cureus.76716

**Published:** 2025-01-01

**Authors:** Emmanouil Koullias, Maria Papavdi, John Koskinas, Melanie Deutsch, Anastasia Thanopoulou

**Affiliations:** 1 Second Department of Internal Medicine, Hippocration General Hospital, National and Kapodistrian University of Athens, Athens, GRC

**Keywords:** fgf analogs, fxr agonists, glp1 receptor agonist, metabolic dysfunction-associated steatohepatitis (mash), metabolic dysfunction-associated steatotic liver disease (masld), ppar agonists, resmetirom, sodium-glucose cotransporter-2 (sglt-2) inhibitors, type 2 diabetes mellitus

## Abstract

Metabolic dysfunction-associated steatotic liver disease (MASLD) affects an ever-increasing part of the global population, affecting millions of individuals worldwide. Despite the progress in the treatment of other liver diseases, there is a scarcity of liver-specific drugs targeting MASLD. In light of that, research has focused both on pipeline drugs targeting multiple different receptors implicated in the pathogenesis of the disease, as well as medications already approved for other indications, that might exert beneficial effects on MASLD. The fact that MASLD is associated with an increased prevalence of obesity and type 2 diabetes mellitus (T2DM) establishes a possible pathway with respect to already available pharmaceutical interventions for this group of patients, such as glucagon-like peptide-1 receptor agonists (GLP-1RAs) and sodium-glucose co-transporter-2 inhibitors (SGLT2-is). Thus, the hitherto at hand, along with the upcoming members of these families, provide much-needed options for our arsenal. This review attempts to explore old and novel dimensions of the pharmaceutical treatment of MASLD in the continuous effort of the medical society to improve patient outcomes.

## Introduction and background

Metabolic dysfunction-associated steatotic liver disease (MASLD) is the most common liver disease on the planet, exhibiting a constantly rising prevalence among various populations and disproportionately affecting those of the Western hemisphere and the Middle East [1]. Differences in prevalence among populations are mainly due to socioeconomic factors, but also, to a degree, due to genetics, with the characteristic example of Latinos, who have a higher prevalence of PNPLA3 leading to increased incidence of MASLD [2]. Of course, the pathogenetic mechanisms of MASLD development are complicated and seem to abide by the theory of “Multiple Hits”. The factors contributing to the disease are multifaceted, including genetic, metabolic, dietary, environmental, and microbiomic influences [3].

Formerly known as non-alcoholic fatty liver disease, the change in nomenclature aims to address the fact of various metabolic dysfunctions associated with the development of the disease, while also establishing the diagnosis with “positive” criteria instead of the “negative” definition, which focuses mostly on the exclusion of overt alcohol consumption as well as other causes of liver disease. The new definition allows for the coexistence of different liver diseases with MASLD. Furthermore, MASLD diagnosis is made when liver steatosis along with one of the following are present: overweight or obesity, prediabetes or type 2 diabetes mellitus (T2DM), hypertension, hypertriglyceridemia and low plasma levels of high-density lipoprotein (HDL) cholesterol.

Naturally, the main risk factors for MASLD development are the presence of obesity, prediabetes or T2DM and dyslipidemia. Additionally, alcohol consumption can lead to a rapid increase in liver fat accumulation, thus promoting steatohepatitis and fibrosis. This group of patients, however, is categorized under the newly formed term “MASLD with increased alcohol intake” or MetALD.

The majority of patients with MASLD are asymptomatic and present with a steatotic liver and persistently mildly elevated aminotransferases. The medical course of the disease is mostly benign. However, a percentage of these patients, over time, are diagnosed with metabolic dysfunction-associated steatohepatitis (MASH), a condition with a high risk for cirrhosis and hepatocellular cancer (HCC) development. MASH also increases the risk of T2DM as well as cardiovascular and chronic kidney disease. Similarly, T2DM disproportionately promotes the development and progression of MASH in patients with liver steatosis. The coexistence of both diseases leads to a markedly increased risk for adverse outcomes, regarding both advanced liver disease, as well as cardiovascular disease. It is a fact that most patients with MASLD perish due to cardiovascular complications (40%), while only about 4-8% of deaths are attributable to liver cirrhosis and/or HCC [1].

From a histological perspective, steatotic liver disease is diagnosed when at least 5% of hepatocytes are infiltrated by lipids but no vacuolar degeneration is present. On the other hand, MASH is diagnosed when at least 5% of hepatocytes are steatotic while at the same time inflammation, as indicated by the presence of vacuolar degeneration, is detected.

Despite the fact that the presence of fibrosis is not necessary for the diagnosis of MASH, the development of fibrosis is the most important prognostic factor regarding adverse endpoints in patients with MASLD, leading to a markedly increased liver-related mortality.

Major steps towards treating MASLD include a drastic change in the patient’s way of life, by adopting both caloric restriction and a regular exercise routine [4], aiming at bodyweight reduction and the improvement of the patient’s physical condition.

Bariatric surgery can be implemented if the lifestyle approach is not enough [5]. Unfortunately, failure is the case most of the time as the majority of patients prove to be unable to lose sufficient weight through diet and exercise alone or cannot maintain any targets they temporarily achieve.

Apart from the above options, the pharmaceutical treatment of MASLD is rapidly gaining ground, with more choices added to our arsenal with each passing year, while many more are currently under study. Novel pharmaceutical approaches attempt to target insulin resistance, de novo lipogenesis, inflammation, oxidative metabolism and fibrogenesis. Examples of such agents include hormonal peptide analogs, most notably glucagon-like peptide 1 (GLP-1), glucose-dependent insulinotropic polypeptide (GIP) and glucagen (GC) receptor agonists, as well as fibroblast growth factor 19 and 21 (FGF-19 and FGF-21) and nuclear receptor ligands, such as peroxisome proliferator-activated receptor α,γ,δ (PPARα,γ,δ), thyroid hormone receptor beta (THRβ) and farnesoid-X receptor (FXR) agonists [6].

## Review

Targeting MASH

Vitamin E

Vitamin E acts as an antioxidant leading to improvement of lipid metabolism regulation [7]. Due to a lack of better choices, this particular agent has been utilized in the recent past for the treatment of patients with MASH, but without T2DM, at a dose of 800 IU daily. The Pioglitazone versus Vitamin E versus Placebo for the Treatment of Nonalcoholic Steatohepatitis (PIVENS) study exhibited improvement of steatohepatitis, without improvement of fibrosis, after 96 weeks of treatment with vitamin E [7]. Furthermore, treatment with vitamin E has been associated with a smaller possibility of decompensation and greater survival without liver transplantation. However, the lack of randomised trials has led to the exclusion of vitamin E from the most recent guidelines [8].

Resmetirom

Resmetirom, a THRβ selective agonist developed specifically for MASH treatment, became recently the first agent to be approved by the FDA with this indication. The phase III study MAESTRO-NASH, which included 966 patients, exhibited improvement of steatohepatitis and fibrosis in 25-30% of the treatment arm compared to 10-14% of the control arm. This was a significant result, especially if we take into account the fact that 60% of participants in the study had advanced liver fibrosis (F3), while only about 5% were at a relatively early stage (F1B) [9]. Regardless, there remains a majority of patients (about 70%) who do not respond satisfactorily to this particular agent. The European Medicines Agency (EMA) decision regarding approval of resmetirom is expected in mid-2025.

Targeting MASH through glycemic and bodyweight control

Metformin remains the backbone of T2DM treatment. It has been linked to increased time until the need for a liver transplant, as well as a reduced risk of HCC and other cancers’ development. However, its effects on liver histology have not proved to be beneficial. Other, second line, treatments of T2DM have exhibited more drastic effects regarding the liver.

PPAR Agonists - Pioglitazone

Pioglitazone is a member of the thiazolidinedione family. It exerts its antidiabetic action through PPARγ activation. It has been shown to improve inflammation in MASH with or without T2DM [7,10], while at the same time improving insulin resistance and the patients’ lipid profile. The PIVENS study exhibited a significant improvement of both liver steatosis and inflammation with the use of pioglitazone [7], while in a study by Cusi et al., long-term treatment of patients with prediabetes or T2DM and MASH led to an improvement of NAFLD Activity Score, as well as a tendency towards fibrosis amelioration [10]. However, long-term use is associated with weight gain, osteopenia and aggravation of congestive heart failure. As is the case with vitamin E, the lack of randomised trials has led to its exclusion from the most recent guidelines, as far as the treatment of MASH is concerned [8].

GLP-1 Receptor Agonists (GLP-1RAs)

GLP-1RAs exert significant effects as far as glucose and lipids metabolism are concerned, while their use is associated with weight loss. These characteristics are advocates of the presence of positive liver effects in patients with MASH, apart from their antidiabetic action.

Liraglutide was shown to improve inflammation in 39% of patients in the relatively small, phase II study, Liraglutide Efficacy and Action in Non-alcoholic Steatohepatitis (LEAN), after 48 weeks of treatment, while decreasing the possibility of fibrosis progression [11]. Liraglutide has also been shown to induce significant body weight loss [12]. The most common side effects include nausea and vomiting.

In the same spirit, in another phase IIb study, with 320 participants treated for 72 weeks, semaglutide administration was associated with significant improvement of steatohepatitis, without fibrosis progression, compared with the control group. The same study exhibited a dose-dependent delay of MASH progression [13]. The phase III study, ESSENCE, regarding the effect of semaglutide on patients with MASH and a fibrosis stage of F2-F3, is currently underway. Furthermore, the combination of semaglutide with firsocostat and cilofexor, both lipogenesis inhibitors, exhibited positive results in phase II studies [14,15]. Regarding weight loss, semaglutide has exhibited impressive results in the clinical trial STEP 1, which included 1961 obese patients. The treatment arm reported a mean loss of 15.3 kg, compared to 2.6 kg in the control group [16].

Tirzepatide, a double GLP-1/GIP-RA, has been shown to be rather effective regarding weight loss in the study SURMOUNT-1, where the treatment group exhibited a relative weight reduction of 20.9%, along with a decrease of total body fat of 33.9% [17]. Similar results were reported by the SURPASS-3 study, where the treated patients exhibited a mean reduction of liver steatosis of 8.09% (in absolute values) [18]. Regarding steatohepatitis, tirzepatide was dose-dependently associated with the resolution of MASH, without worsening of fibrosis, in 44%-62% of treated patients in the phase II study, SYNERGY-NASH [19]. In the same study, the percentage of patients that received tirzepatide and exhibited an improvement of at least one fibrosis stage, without worsening of MASH, was between 51% and 55%, compared to 30% in the control group [19].

Survodutide, another double agonist (GLP-1/GC-RA), was shown in the phase II study NCT04771273, which included 293 patients with MASH and F1-F3 fibrosis, to lead to improvement of MASH, without fibrosis progression, in 43-62% of the treatment arm, compared with 14% of the control group. At the same time, a relative liver fat fraction reduction greater than 30% was achieved in 57%-67% of the patients receiving survodutide, compared with 14% in the control group, after 48 weeks of treatment [20]. Survodutide was also shown to induce significant dose-dependent bodyweight loss and HbA1c reduction, similar to or even better than with semaglutide 1mg/week, after 16 weeks of treatment, in the phase II study NCT04153929 [21]. In another phase II study (NCT04667377), survodutide achieved a relative weight reduction of almost 15%, compared with 2.8% in the placebo group [22]. The main side effects of survodutide were related to gastrointestinal events and were mostly of mild or moderate severity.

Cotadutide (GLP-1/GC-RA) has been tested in several phase II studies in patients with T2DM and obesity, where it showed satisfactory results in terms of glycemic control and weight loss [23]. Also, in the randomized phase II trial, PROXYMO, which enrolled 74 non-cirrhotic patients with MASH, demonstrated significant improvement in liver steatosis [24].

Efinopegdutide (GLP-1/GC-RA) has been shown to improve aminotransferase levels, hepatic steatosis, markers of fibrosis and body weight in patients with MASLD, obesity and T2DM in a phase IIA study (NCT04944992) with 145 participants. More specifically, efinopegdutide at 10 mg weekly appeared to induce a relative reduction in the hepatic fat fraction of 72.7% versus 42.3% induced by semaglutide at 1 mg weekly, with the effect on hepatic steatosis being independent of BMI reduction, as it was also observed in patients with a small change in their bodyweight during the study [25].

Sodium-Glucose Co-transporter 2 Inhibitors (SGLT2-is)

SGLT2-is are drugs that contribute to glycemic regulation, while simultaneously promoting weight loss and exhibiting a multitude of other effects. Their contribution seems to extend to non-diabetic patients, as a result of which, they are also approved for administration to patients with heart failure and chronic kidney disease. The antioxidant and anti-inflammatory effects of this group make it a candidate for the treatment of patients with MASLD. However, studies that have addressed the results of these drugs on the liver are generally small and, mostly, do not include histological evaluation.

Characteristic examples of these studies include the one by Latva-Rasku et al., where the addition of dapagliflozin to metformin and/or a DPP-4 inhibitor contributed to the reduction of liver and visceral fat in obese T2DM patients, after eight weeks of treatment, as measured by magnetic resonance imaging-proton density fat fraction (MRI-PDFF) [26], as well as the study by Kahl et al., where the addition of empagliflozin 25mg to T2DM patients with good glycemic control resulted in a relative reduction of liver fat by 22%, as measured by volume-selective proton MRS (1H-MRS), and a reduction of body weight by 2.5kg, after 24 weeks of treatment [27]. Additionally, worth mentioning is the study by Cusi et al., in which administration of canagliflozin 300 mg daily led to 38% of patients exhibiting a weight reduction of at least 5% and a relative reduction in liver fat by of at least 30%, after 24 weeks of treatment, compared to 7% of control group patients [28].

Several other studies in humans have confirmed the existence of positive effects regarding the reduction of liver steatosis, stiffness and fibrosis. However, the majority of them included a relatively small number of participants and were defined by variable inclusion criteria and follow-up periods.

Meta-analyses have shown that the improvement in liver fat seen with SGLT2-is treatment appears to be primarily due to weight loss and glycemic control [29,30]. These data, however, cannot fully justify the observed amelioration of steatosis, suggesting that there are further factors involved, regarding the hepatoprotective properties of SGLT2-is in MASLD patients [27].

As observed in preclinical models, it appears that treatment with these drugs generally improves insulin resistance, decreases intracellular free fatty acids and triglyceride accumulation, as well as total cholesterol, by reducing the expression of de novo adipogenesis genes. In addition, they reduce the hepatic uptake of fatty acids, while at the same time, they promote the expression of genes regulating fatty acid β-oxidation [31]. Furthermore, the beneficial effects of SGLT2-is appear to involve the diminishment of liver inflammation. Possible mechanisms include increasing Nrf2 expression [32], decreasing NFκB (nuclear factor kappa-light-chain-enhancer of activated B cells) and MCP-1 (monocyte chemoattractant protein-1) expression, the inhibition of the IL17/IL23 axis [33], the reduction of the expression of inflammatory cytokines in the liver, such as TNF-α, IL-1β, IL-18 and IL-6, [34], as well as the promotion of antioxidant and antiapoptotic actions, such as reducing hepatic production of reactive oxygen species (ROS) and thiobarbituric acid reactive substances (TBARS), and caspase-3 levels [34,35]. Antifibrotic properties have also been observed, through the reduced expression of transforming growth factor beta (TGF-β) [31,34] and tissue inhibitors of metalloproteinases (TIMPs) [31].

In conclusion, SGLT2-is decrease blood glucose and insulin levels, especially in diabetic patients. This in turn leads to a large reduction in hepatic de novo lipogenesis. SGLT2-is also appear to act on pancreatic α-cells, leading to an increase in glucagon secretion [36], which promotes β-oxidation of fatty acids, and consequently, reduces liver triglycerides and improves liver steatosis [36,37]. These effects are enhanced by the antioxidant activity of these drugs, which is due to the reduction of both glucotoxicity and ROS as well as the induction of antioxidant enzymes, such as superoxide dismutases and glutathione peroxidases [38].

SGLT2-is are often co-administered with GLP-1RAs, since both drug families appear to induce weight loss and provide hepatoprotective and cardioprotective benefits, in addition to glycemic control. Table 1 summarizes the apparent effects of currently circulating drugs on the liver.

**Table 1:** Drugs and their beneficial effects on the liver MASH: Metabolic dysfunction-associated steatohepatitis; MASLD: Metabolic dysfunction-associated steatotic liver disease; T2DM: Type 2 diabetes mellitus

Drug	Targeted patients	Liver effects
Vitamin E	MASH without T2DM/cirrhosis	Improvement of steatosis, steatohepatitis without improvement of fibrosis
Pioglitazone	MASH with/without T2DM	Improvement of steatosis and steatohepatitis, maybe fibrosis
Liraglutide	MASH with T2DM without cirrhosis, MASH with obesity	Improvement of steatosis, steatohepatitis without improvement of fibrosis
Semaglutide	MASH with T2DM without cirrhosis	Improvement of steatosis, steatohepatitis without improvement of fibrosis
Dulaglutide	MASLD with T2DM	Improvement of steatosis, steatohepatitis without improvement of fibrosis
Tirzepatide	MASLD with T2DM or obesity	Improvement of steatosis and steatohepatitis
Empagliflozin	MASLD with T2DM or/and heart failure	Improvement of steatosis
Dapagliflozin	MASLD with T2DM or/and heart failure	Improvement of steatosis
Canagliflozin	MASLD with T2DM	Improvement of steatosis

Pipeline medications targeting MASH

FXR Agonists

Obeticholic acid is an FXR agonist with antifibrotic properties. In the phase III study REGENERATE, which involved 931 participants, the proportion of pre-cirrhotic MASH patients (F2-F3) under treatment with obeticholic acid, who achieved a histological regression of fibrosis of at least one grade, without worsening of steatohepatitis, was 22.4%, versus 9.6% in the control group [39]. Accordingly, it appeared that a lower proportion of patients treated with obeticholic acid developed cirrhosis at the end of the study compared to the control group. However, after the New Drug Application was rejected by the FDA, the study was prematurely terminated by the corresponding pharmaceutical company. This drug is so far the only approved second-line treatment for non-responder primary biliary cholangitis (PBC) patients.

Cilofexor is another FXR agonist which has been demonstrated to reduce liver steatosis in a phase II trial [40], after 24 weeks of treatment and has also been utilized, without success, on patients with primary sclerosing cholangitis and primary cholangitis. Administration of cilofexor in combination with firsocostat and semaglutide has demonstrated beneficial effects as far as liver steatosis, inflammation activity and fibrosis are concerned [14,15]. However, phase III studies have not yet been announced.

Tropifexor has also exhibited beneficial effects regarding hepatic fat fraction and bodyweight reduction after 12 weeks of treatment in the phase II trial NCT02855164 [41].

Fibroblast Growth Factor (FGF) Analogs

Pegozafermin is a long-acting glycopegylated analog of FGF21 that is being studied for potential use in the treatment of MASH and severe hypertriglyceridemia. It is administered subcutaneously. Pegozafermin was administered in the phase IIb ENLIVEN study, which enrolled 219 patients with MASH and F2-F3 stage fibrosis. The results demonstrated that treatment for 24 weeks with the dose of 15 mg or 30 mg weekly and 44 mg every two weeks led to an improvement in fibrosis by at least one grade, without worsening of steatohepatitis, in 22%, 30% and 27% of patients in the three treatment groups, versus 7% in the control group. Resolution of MASH (without worsening of fibrosis) was respectively observed in 37%, 23% and 26% of patients, compared to 2% in the control group. The most common side effects were nausea and diarrhoea [42]. A phase III study, which will focus on patients with MASH with compensated cirrhosis (F4), will further evaluate pegozafermin (ENLIGHTEN-cirrhosis), by assessing fibrosis regression after 24 months of treatment.

Efruxifermin is another long-acting FGF21 analog. The phase IIb study HARMONY enrolled 128 patients with MASH and grade F2-F3 fibrosis who received either efruxifermin (28 mg or 50 mg subcutaneously weekly) or placebo for 24 weeks. Improvement of fibrosis by at least one grade (without worsening of steatohepatitis) was observed in 20% of patients in the placebo group, 39% of patients receiving 28 mg efruxifermin, and 41% of patients receiving 50 mg efruxifermin. The most frequent side effects were diarrhoea and nausea [43]. Despite that, the phase IIb SYMMETRY study failed to exhibit a significant improvement in fibrosis after 36 weeks of treatment. Currently, three phase III studies are underway, targeting to assess the effect of efruxifermin on patients with MASLD and fibrosis of varying degrees.

Aldafermin is an FGF19 analog. It has exhibited benefits regarding liver steatosis reduction (greater than >30%), as well as improvement of aminotransferase levels and the Enhanced Liver Fibrosis (ELF) score, without, however, showing a statistically significant benefit with respect to fibrosis, in the studies conducted so far [44,45].

Stearoyl-CoA Desaturase-1 (SCD1) Inhibitors

Aramchol is a partial inhibitor of hepatic SCD1. In the double-blind, phase IIb study ARREST, 247 patients with MASH were randomized to receive either placebo or 400 mg or 600 mg of aramchol daily. The primary endpoint of the study was the reduction in hepatic steatosis, as measured by magnetic resonance imaging. Secondary endpoints included improvement in liver histology and alanine transaminase (ALT). Despite the improvement in steatosis, statistical significance was not achieved for this endpoint. However, improvement of steatohepatitis without worsening of fibrosis was achieved in 16.7% of patients receiving 600 mg aramchol versus 5% of those in the placebo group. Simultaneously, improvement of fibrosis by at least one stage, without worsening of steatohepatitis, was achieved in 29.5% of patients, who received 600 mg aramchol, compared to 17.5% of the control group [46]. Despite the failure to meet the primary endpoint, the achievement of histological improvement led to the decision to conduct a phase III study, which is currently underway (AMOR study).

PPAR Agonists

Lanifibranor is a pan-PPAR agonist, which was shown to improve both steatohepatitis and fibrosis compared to the control group in the phase IIb study NATIVE (247 participants with or without T2DM) [47]. A particularly important element of this study was the large proportion of participants with significant fibrosis (F2 or F3 stage in 188 out of 247 patients). Therefore, this drug will be further evaluated in the ongoing phase III NATiV3 study. This study aims to assess lanifibranor’s effect on MASH resolution and fibrosis improvement, as well as its effect on delaying disease progression.

Saroglitazar is a dual PPAR-α/γ agonist. It promotes glycemic regulation and lipid profile improvement, while also appearing to exert a beneficial effect on MASH, by improving hepatic steatosis and perhaps, fibrosis, independently of body weight reduction. This substance is approved in India for use in patients with stage F1-F3 fibrosis [48].

Medicines without effect on MASH

Several drugs have been studied over the past decades for use in the treatment of MASH. Despite sometimes initially promising results, subsequent studies have not confirmed any significant benefit with respect to most of them [49]. Typical examples are metformin, where any MASH benefit was attributed to weight loss and lifestyle change, and arctodeoxycholic acid, which exhibited an improvement in liver biochemistry in pilot studies, but did not confer clinical benefit in randomized trials. Silymarin also did not appear to improve MASH, despite the presence of data suggestive of improvement in fibrosis. Accordingly, both fenofibrate and ezetimibe appeared to lag in improving liver steatosis and MASH. Finally, neither DPP-4 inhibitors, nor acarbose and insulin, seem to benefit steatohepatitis. Recently abandoned pipeline drugs, that were thought to have a beneficial effect on MASH and/or fibrosis in early stages, but have not successfully passed the test of phase III clinical trials, include selonsertib, elafibranor, pegbelfermin, belapectin, simtuzumab, emricasan, MSDC-0602K and cenicriviroc [49].

Discussion

MASLD is the most common cause of liver disease worldwide, with a very high prevalence among T2DM patients. As mentioned above, dietary interventions, lifestyle changes and regular exercise usually bring temporary results, while most of the time they remain inapplicable by patients, given the time, effort and money required.

However, not for lack of trying, there is a scarcity of drugs for the treatment of MASLD. Despite the resounding successes in treating viral hepatitis over the last decade, the results regarding MASH are humbling. This could be attributed to the diverse, not fully understood, pathogenic processes driving the disease, along with faults in the design of the relevant studies or the interpretation of their results. Due to the pressure of the unmet need for developing MASH treatment options, many substances could be advanced through phase II to phase III, after exhibiting supposedly promising results that are in fact, equivocal. The achievement of a single endpoint may translate to conducting a much larger trial on a drastically different population, ultimately leading to failure. Furthermore, MASLD is a disease that progresses over decades, through various phases and the “correct” phase in which a specific agent may be efficacious is hard to determine. A drug that achieves reversal of fibrosis in an animal model will not necessarily do so in humans that have liver fibrosis for an indeterminate, but certainly longer, amount of time. To add insult to injury, the strict criteria of fibrosis improvement and MASH resolution required in MASH studies are subject to intra-reader variability of opinion leading to unknown extents of fallacies in respect to both inclusion of suitable candidates and proper assessment of the experimental agent’s or the placebo’s effect. In addition to these, underpowered studies, short-term follow-up periods and trial durations as well as insufficient optimization of drug doses in the preliminary phases could add further aspects to the problem [49].

Given the absence of drug options aimed at treating MASLD, with the exception of the recently approved resmetirom and the older controversial options of vitamin E and pioglitazone, it was expected that research interest would shift, beyond the discovery of new substances, encompassing already approved drugs, originally aimed at an indication other than MASLD. Those medicines’ effects on MASLD could be more easily studied in the context of the treatment of these particular patients' comorbidities. Examples of such drugs are, among others, aspirin, DPP-4 inhibitors, metformin, statins, fenofibrate, and ezetimibe, with variable, but not spectacular, results in each case. The approval of the newest antidiabetic medications, characterized by a plethora of effects other than glycemic control, has breathed new life into the treatment of patients with MASLD and T2DM, offering much-needed additional options to our arsenal.

From the available options though, the antidiabetic medications from the GLP1-RAs and SGLT2-is families seem to be the most readily available, offering patients with MASLD and T2DM some rather accessible treatment choices.

Significant examples of clinical trials supporting this approach, as already mentioned, include those that utilized liraglutide and semaglutide, the administration of which appeared to exert a beneficial effect both on liver inflammation and, mainly, on steatosis. Regarding dulaglutide, an open-label study by Kuchay et al. (D-LIFT) reported an improvement in a hepatic fat fraction [50], while in another study no such effect was exhibited [51]. Similar examples of SGLT2-is administration, apart from those already mentioned above, include the studies by Kuchay et al. [52], Chehrehgosha et al. [53] and Taheri et al. [54], which showed a benefit in hepatic steatosis from the administration of empagliflozin, the studies by Eriksson et al. [55] and Kurinami et al. [56], which showed a similar benefit from the administration of dapagliflozin, as well as the study by Nishimiya et al. [57], in which canagliflozin was administered.

A common feature of most of these studies is the coexistence of T2DM and MASLD in the participants, but also importantly, the short duration of follow-up, which usually lasted from three to six months. Relatively recently, studies of longer duration of 48 to 72 weeks have been published, assessing mainly semaglutide’s effect on steatohepatitis [13] and less on steatosis.

Both of these families have been shown to exert cardioprotective and nephroprotective effects, making them a valid choice even in the case of the proliferation of liver-specific alternative therapies in the near future. The addition of the recently approved resmetirom or the understudy lanifibranor, obeticholic acid, aramchol, etc. is not likely to affect the position gradually established by SGLT2-is and GLP-1RAs in the treatment of patients with MASLD. An additional element in favour of this argument is further results of studies published in the last years, which demonstrate a reduced risk of portal hypertension, decompensation and HCC in patients treated with GLP1-RAs or SGLT2-is [58-60].

Given their effectiveness and the population, to which they are basically addressed, numbering in the hundreds of millions of patients, the scope for economies of scale, competition between pharmaceutical companies and further development of related, even more effective, or less frequently administered, substances is expected to lead to easier access and significantly reduced treatment costs in the future, making targeting SGLT2 and the GLP1 receptor a cornerstone in the management of patients with MASLD and comorbidities.

## Conclusions

MASLD is and will remain, for the foreseeable future, the major liver disease affecting the global population. The spectrum of the disease is broad, while its progress is usually slow and governed by multiple, not clearly defined, pathogenetic mechanisms. The difficulty of pinpointing the duration and stage of the disease of individual patients raises further obstacles in designing and carrying out proper clinical trials that would be able to assess the effects of each candidate substance. This translates into further hardship in the continuous effort of establishing treatment options for MASLD, leading to a paucity of choices regarding these patients, with resmetirom being the only agent to have been approved after years of failed studies and multiple aborted pipeline medications.

The already-approved medications currently being used for the treatment of obesity and T2DM seem to benefit MASLD, at least with respect to steatosis and steatohepatitis, due to the intensification of glycemic control, induction of weight loss and possibly, due to a series of other molecular actions. GLP-1RAs and SGLT2-is are already being prescribed to millions of patients and their effect on MASLD should be further explored and exploited. Their accessibility, relatively low cost and established safety, along with the advantages they offer, with respect to heart and kidney disease, make them valid options until the arduous process of clinical trials finally gives birth to more liver-specific drugs.
